# Physical Activity and Mental Health of Patients with Pulmonary Hypertension during the COVID-19 Pandemic

**DOI:** 10.3390/jcm9124023

**Published:** 2020-12-12

**Authors:** Carolin Leoni Dobler, Britta Krüger, Jana Strahler, Christopher Weyh, Kristina Gebhardt, Khodr Tello, Hossein Ardeschir Ghofrani, Natascha Sommer, Henning Gall, Manuel Jonas Richter, Karsten Krüger

**Affiliations:** 1 Department of Exercise Physiology and Sports Therapy, Institute of Sports Science, Justus Liebig University Giessen, 35394 Giessen, Germany; Carolin.Dobler@sport.uni-giessen.de (C.L.D.); christopher.weyh@sport.uni-giessen.de (C.W.); kristina.gebhardt@sport.uni-giessen.de (K.G.); 2 Nemolab, Institute of Sports Science, Justus Liebig University Giessen, 35394 Giessen, Germany; g51130@uni-giessen.de; 3 Department of Psychotherapy and Systems Neuroscience, University of Giessen, 35394 Giessen, Germany; Jana.Strahler@psychol.uni-giessen.de; 4 Bender Institute of Neuroimaging, Justus Liebig University Giessen, 35394 Giessen, Germany; 5 Department of Internal Medicine, Universities of Giessen and Marburg Lung Center (UGMLC), Member of the German Center for Lung Research (DZL), Excellence Cluster Cardio-Pulmonary Institute (CPI), Justus-Liebig University, 35394 Giessen, Germany; Khodr.Tello@innere.med.uni-giessen.de (K.T.); ardeschir.ghofrani@innere.med.uni-giessen.de (H.A.G.); Natascha.Sommer@innere.med.uni-giessen.de (N.S.); Henning.gall@innere.med.un-giessen.de (H.G.); Manuel.Richter@innere.med.uni-giessen.de (M.J.R.)

**Keywords:** resilience, active lifestyle, stress levels, infection control measure, self-quarantine

## Abstract

The aim of the study was to analyze the effect of personal restrictions on physical activity, mental health, stress experience, resilience, and sleep quality in patients with pulmonary hypertension (PH) during the “lockdown” period of the COVID-19 pandemic. In total, 112 PH patients and 52 age-matched healthy control subjects completed a questionnaire on the topics of physical activity, mental health, resilience, and sleep quality. PH patients had significantly lower physical activity, mental health, and sleep quality compared to age-matched healthy controls. Physical activity positively correlated with mental health and sleep quality in the PH group. Mental wellbeing and life satisfaction could be predicted by total physical activity, sleep, stress level, and resilience. PH patients appeared as an especially vulnerable group, demanding interventions to promote an active lifestyle and protect mental health in these patients. This could be helpful in counseling on how to carry out physical activity while maintaining infection control.

## 1. Introduction

Pulmonary hypertension (PH) is a multifactorial chronic pulmonary disease which is defined by an elevated mean pulmonary arterial pressure, which untreated eventually can lead to right heart failure and death [1]. Depending on the clinical classification and risk stratification PH patients have quite a different prognoses, treatment options, and impairment in daily life [2]. Patients with PH experience symptoms such as shortness of breath, exertion, fatigue, chest pain that restrict physical activity, which in turn impairs quality of life and favors mental disorders such as depression [3,4,5,6]. Accordingly, PH patients experience a high degree of functional limitations, which was shown to be almost comparable to those reported by cancers patients [7].

In January 2020, the outbreak of COVID-19 was declared as a “Public Health Emergency of International Concern” by the WHO. On January 27th, the first COVID-19 infection in Germany was detected. By end of March 2020, the COVID-19 was classified as a pandemic. Since “socio-physical distancing” is seen as one of the most effective strategies to reduce the number of infections, Robert-Koch Institute (RKI) called on the population in Germany to keep their distance from other people [8]. As elderly and patients with specific risk factors and pre-existing diseases are at a higher risk of severe COVID-19 course of disease and mortality [8], these groups, including PH patients, were prompted to be particularly careful to reduce individual infection risks. One can therefore assume that many persons at risk stayed at home, reduced their physical activity, and performed a social distancing to protect themselves.

Recent studies indicate that common reactions to the COVID-19 pandemic and the protection measures are elevated levels of anxiety [9,10,11], depression [12,13], and stress [14]. Being a woman and having a (chronic) disease [15] are factors associated with stronger mental burdens during COVID-19 relate to shock and lockdown measures amongst others. Those identifying themselves as a high-risk group when being infected with COVID-19 also showed higher levels of anxiety, depression, and stress symptoms [10]. Furthermore, it seems reasonable that high-risk patients are particularly careful and isolate themselves, which ultimately may result in physical deconditioning [12,13,14]. Decreased exercise capacity and emotional difficulties such as anxiety, depression, and stress correlate with a negative HRQoL of patients with pulmonary arterial hypertension (PAH) [6]. Research also shows that this relationship works in both directions with PH symptoms reinforcing stress and anxiety [10]. Data suggests that PH patients might be very susceptible to mental impairment due to pandemic restrictions and protection measures. Remaining physically active though has been associated with better mental health scores during this pandemic [13,16]. Whether this also applies to PH patients is currently unknown. Related knowledge would however be of utmost importance to better understand risk factors of lockdown measure-related vulnerabilities and possible therapeutic approaches.

On this background, the present study aimed to investigate the quality of life of PH patients during the first weeks of the lockdown period of the COVID-19 pandemic in Germany. More specifically, associations between mental health, stress experience, physical activity, sleep quality, and sociodemographic factors were analyzed. As the individual’s resilience (i.e., adapting, managing, and negotiating adversity) [17] is another important factor supporting mental health and wellbeing as it for example moderates physical activity effects, we also assessed resilience. We analyzed whether and to which degree resilience, physical activity, stress experience, and sleep behavior determined mental health issues. We further explored whether the subjective stress level is related to the perceived mental well- or illbeing of PH patients compared to an age and education-matched control group. We hypothesize that PH patients experience a stronger reduction of physical activity, sleep quality, and resilience as well as an increased subjective stress experience during the pandemic than healthy subjects, which is at the same time associated with a higher psychological burden.

## 2. Methods

### 2.1. Subjects Characteristics

In this cross-sectional study, subjects diagnosed with pulmonary hypertension were compared to a healthy control group concerning to their mental health represented by WHO-5, PHQ-4, L-1, physical activity, resilience, stress experience, and sleep quality during the COVID-19 associated lockdown. The control group was matched by age.

Subjects of the PH group had to have their residence inside of Germany and a medical diagnosis of PH. To be eligible for the control group, subjects must not have had a confirmed infection or symptoms of COVID-19, any comorbidities, or a residence outside of Germany. Only subjects declaring consent were included in this study.

PH patients of the University Hospital of Gießen and Marburg (UKGM) were directly contacted. The questionnaire was promoted during online consultation hours of the UKGM and in PH support groups (https://pulmonale-hypertonie-selbsthilfe.de/). If necessary, the questionnaire was sent to patients per mail due to lacking access to the internet and was entered into the online survey by hand.

In total, 251 PH patients followed the invitation to participate in the survey. There were 139 cases that were excluded, 111 of those for not completing the questionnaire, 25 for reporting to have no medical diagnosis of PH, and three because their residence was outside of Germany. This left us with questionnaires of 112 subjects being valid for the PH group. The mean age in the PH group was 54.4 ± 14.0 years with 77.7% being female. The average number of comorbidities was 2.6 ± 2.0. 52 healthy subjects were recruited into the control group in dependence of their eligibility criteria and age matching. The mean age of the control group was 52.3 ± 8.9 years with 67.3% being female. The sociodemographic characteristics of both groups are presented in Table 1.

### 2.2. Questionnaire and Outcomes

The survey contained several questions and validated questionnaires to evaluate study variables. In the beginning, sociodemographic data such as age, gender, relationship status, living situation, the number of residents per household, educational level, occupational status, worries about health, and satisfaction with sports behavior were gathered. The living situation covered the living area and the availability of a garden or a balcony. The number of residents per household was divided into the total number of residents and those younger than 18 years. The category comorbidity included sub-categories of comorbidities (apart from PH for the PH group). Every sub-category could be answered with “No”, “Yes, medical diagnosis”, or “Yes, self-assessment”, although a self-assessed diagnosis was counted as a “No” in the analysis. The number of “Yes, medical diagnosis” was counted and summed up to create the variable “Comorbidities”. Possible SARS-CoV-2 infection or symptoms were also evaluated.

Physical activity data were collected through items three to six of the BSA-F which was shown to be a valid tool for the measurement of physical activity and sports behavior [18]. The outcome scores were *total physical activity*, *the activity of daily living*, *sports activity,* and *climbing stairs*. The variable *total physical activity* is a sum of the *activity of daily living* and *sports activity*. The amount of different physical activity, exercise and sports modalities are calculated by multiplying the duration and frequency of the corresponding items and then summating them. The examined period for all scores was four weeks in the PH group. The scores *total physical activity*, *activity of daily living* and *sports activity* were converted into the unit minutes/week as designated and were thereby made comparable. The item *climbing stairs* was converted into unit floors/week.

Mental health was assessed by using the WHO-5 Well-Being Index (WHO-5), the Patient Health Questionnaire-4 (PHQ-4), the L-1-scale, one question on perceived stress, and four items measuring state resilience [19]. The 5-item WHO-5 scale, which assesses subjective wellbeing but is also a valid tool for the screening of depression [20], was converted into a score ranging from 0 to 100 for comparison with other studies. Zero represents the worst mental health and a score ≤50 was used as sign for depression. Another valid and reliable score for the investigation of depression and anxiety in the general population is the PHQ-4 [3]. The PHQ-4 was added up to create a single score ranging between 0 and 12 with 0 indicating good mental health. The PHQ-4 consists of the Patient Health Questionnaire-2 (PHQ-2) measuring depression and the Generalized Anxiety Disorder 2 (GAD-2) quantifying anxiety. The overall life satisfaction was measured by the valid and reliable eleven-point L-1-scale [21] with 0 indicating no satisfaction and 10 representing a strong overall life satisfaction. Resilience was measured by four eight-point Likert-scales that were then averaged to a total score [19]. The subjective stress-level was assessed by a further eight-point Likert-scale rating the statement “I feel stressed out”.

Sleep data were divided into general sleep quality and current sleep quality. Both scores were measured through a ten-point Likert-scale with 1 indicating bad sleep quality and 10 indicating excellent sleep quality.

### 2.3. Statistical Analysis

First, descriptive statistics were performed for both groups. Prior to this, we z-standardized all variables.

In the next step, we tested all observed variables regarding their distribution features. As none of the variables were normally distributed, the Mann–Whitney-U-Test was used to identify differences between the PH and the control group regarding mental health scores (WHO-5, PHQ-4, L-1), physical activity (*total physical activity*, *the activity of daily living*, *sports activity*, *climbing stairs*) resilience, stress experience, and quality of sleep.

We further used Spearman’s rank correlation coefficients to determine the relationship between mental health (as indicated by WHO-5, L-1, and PHQ-4), physical activity (*total physical activity*, *the activity of daily living*, *sports activity*, *climbing stairs*), resilience, feelings of stress, and sleep quality for the PH and the control group separately.

Lastly, we analyzed the impact of resilience, total physical activity, sleep quality and feelings of stress as potential predictors of mental health using multiple regression analyses for the PH group as well as over both groups. We, therefore, further added four product terms to the model to test for an interaction between total physical, resilience, stress, sleep, and group membership. SPSS 22 was used for statistical analysis and partly for graphical illustration. For further illustration of the data, we used Python 3. *p* values < 0.05 will be considered significant. Bonferroni-correction was used to correct for multiple comparisons.

## 3. Results

### 3.1. Mental Health Indicated by WHO-5, PHQ-4, and L-1

For the PH group, the level of mental health was represented through the WHO-5 scoring 11.9 ± 5.6, the PHQ-4 being 3.9 ± 3.0, and the L-1 being 5.6 ± 2.7 on average. In the present cohort, a prevalence of depression ranged from 36.4% and 53.6% derived from the WHO-5 score (score less than or equal to 50 as depressed), the PHQ-2 yellow flag (score greater than or equal to 3), and PHQ-2 red flag. Symptoms of anxiety were found in about one-third of the PH patients. The assessment of mental health revealed significantly lower life satisfaction (L1), well-being (WHO-5) as well as increased levels of depression and anxiety (PHQ-4) in the PH group regarding WHO-5, PHQ-4 and its subscores PHQ-2 and GAD-2 and L-1 (all *p* < 0.001) (Table 2).

### 3.2. Resilience, Stress Experience, and Sleep Quality

**Figure 1:**
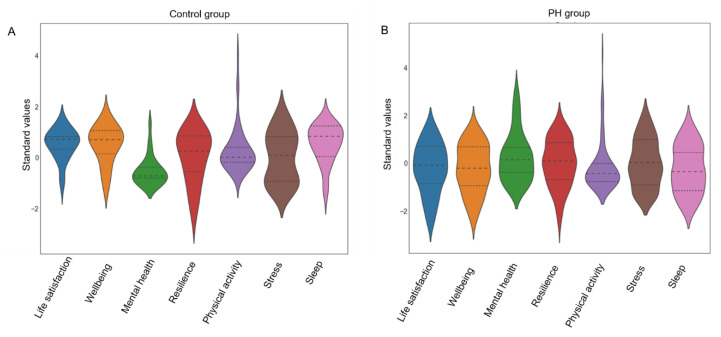
Z-standardized values of mental health variables. (**A**): z-standardized values of life satisfaction, mental wellbeing, mental health, resilience, total physical activity, stress experience, and sleep habits of healthy controls during the lockdown. The dashed inner line of each violin graph reflects the quartiles of each health variable. (**B**): z-standardized values of life satisfaction, mental wellbeing, mental health, resilience, total physical activity, stress experience and sleep habits of PH patients. The dashed inner line of each violin graph reflects the quartiles of each health variable.

The level of resilience was rated on average 4.1 ± 1.6, subjective stress experience was rated on average 2.9 ± 2.1 in the PH group. The general sleep quality was rated as 5.7 ± 2.4 on average, while the current sleep quality was only 5.3 ± 2.5. The level of resilience as well as the subjective stress experience in the PH group did not differ significantly compared to the control group. The general and current sleep quality were both significantly lower in the PH group compared to the control group (both *p* < 0.001). The estimation of one’s living standard showed a statistically significant increased score in the PH group regarding the item “worries about health” and a decreased score in the PH group regarding the item “satisfaction with sports behavior” (*p* < 0.001). Results are depicted in Figure 1 and Table 2.

### 3.3. Physical Activity

The average *total physical activity* of the PH group was 684.3 ± 954.3 (min/week), whereby 551.4 ± 816.9 (min/week) were accounted for by the *activity of daily living* and 129.4 ± 219.8 (min/week) for *sports activities*. The mean score of *climbing stairs* was 14.3 ± 23.0 (floors/week). All modalities of physical activity were significantly lower compared to the control group (all *p* < 0.001, Table 2).

### 3.4. Associations between Subjects’ Characteristics, Physical Activity, Mental Health (Reflected by L-1, WHO-5, PHQ-4), Resilience, and Stress Experience in PH Patients

The four activity indices were correlated with the mental health scores, resilience, stress experience, sleep quality, and sociodemographic data in the PH group (Table 3 for a detailed description). *Total physical activity* showed a significant positive correlation with WHO-5 (r = 0.26, *p* = 0.016), L-1 (r = 0.30, *p* = 0.005), general sleep quality (r = 0.31, *p* = 0.003), and current sleep quality (r = 0.25, *p* = 0.018). The subcategory *activity of daily living* significantly correlated with WHO-5 (r = 0.33, *p* = 0.002), L1 (r = 0.33, *p* = 0.001), general (r = 0.33, *p* = 0.001), resilience (r = 0.236, *p* = 0.027), and acute sleep quality (r = 0.32, *p* = 0.002). *Sports activity* though only showed a significant correlation with age (r = -0.23, *p* = 0.020) and educational level (r = 0.27, *p* = 0.007) but not with any of the self-report measures. Significant correlations regarding *climbing stairs* were found with WHO-5 (r = 0.33, *p* = 0.002,), PHQ-4 (r = 0.23, *p* = 0.031), L-1 (r = 0.31, *p* = 0.003), general sleep quality (r = 0.39, *p* < 0.001), resilience (r = 0.312; *p* = 0.004), and current sleep quality (r = 0.32 *p* = 0.002). These associations were not found in the control group. All correlations for the PH group can be found in Table 3.

### 3.5. Regression Analysis: Prediction of the Mental Health by Resilience, Stress Experience, and Sleep Quality during the Lockdown

In a first step, we calculated regression models for the PH group only to investigate whether life satisfaction, mental well- and illbeing (represented by L1, WHO-5, and PHQ-4) could be predicted by total physical activity, sleep, stress, and resilience.

**Figure 2:**
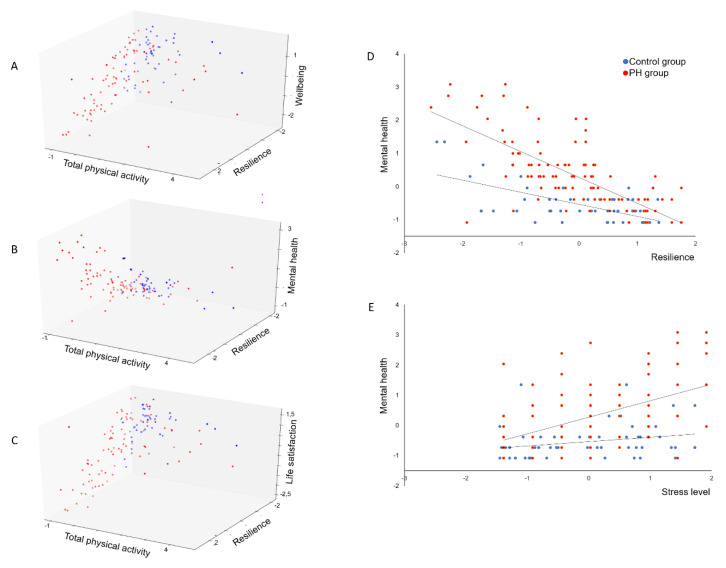
Associations between mental health dimensions, daily activity, and resilience for PH and control group. (**A**): Association between total physical activity, resilience, and wellbeing for PH and control group. (**B**): Association between total physical activity, resilience, and mental health for PH and control group. (**C**): Association between total physical activity, resilience, and life satisfaction for PH and control group. (**D**): Association between resilience and mental health for PH and control group. (**E**): Association between stress and mental health for PH and control group.

The present data revealed that life satisfaction of PH patients is significantly predicted by sleep quality (B = 0.327, beta = 0.321, *p* = 0.001), total physical activity (B = 0.190, beta = 0.178, *p* = 0.012), stress experience (B = −0.215, beta = −0.184, *p* = 0.017), and resilience (B = 0.465, beta = 0.438, *p* < 0.001). The total variance explained by the full model as a whole was *R*^2^ = 0.632, *F*(4, 82) = 36.260, *p* < 0.001. For psychological wellbeing, we found significant associations for sleep quality (B = 0.248, beta = 0.264, *p* = 0.001), stress experience (B = −0.195, beta = −0.181, *p* = 0.006), and resilience (B = 0.579, beta = 0.591, *p* < 0.001) in the PH group. The total variance explained by the model as a whole was *R*^2^ = 0.739, *F*(4, 80) = 57.596, *p* < 0.001. With regard to mental health, we found significant associations for sleep quality (B = −0.182, beta = −0.175, *p* = 0.03), stress experience (B = 0.348, beta = 0.293, *p* < 0.001), and resilience (B = −0.601, beta = −0.555, *p* < 0.001) in the PH group. The total variance explained by the model as a whole was *R*^2^ = 0.682, *F*(4, 81) = 44.379, *p* < 0.001. These data revealed that especially resilience, sleep quality, and stress experience are relevant predictors of mental health outcomes and wellbeing. The patients’ total physical activity only seem to impact variables related to life satisfaction (Figure 2A–C).

When considering the total sample and including the group variable in order to reveal differences between the PH group and healthy controls, linear regression analysis revealed that the mental health issues and mental wellbeing could be predicted by the diagnosis of PH, resilience, stress experience, total physical activity, and the current sleep quality. Total physical activity level had an albeit smaller impact on mental wellbeing.

Regarding life satisfaction, results showed that the L1-score was significantly lower for PH patients compared to healthy controls (B = −0.482, beta = −0.226, *p* < 0.001). Moreover, increases in L1-scores correlated significantly with increases in resilience (B = 0.444, beta = 0.449, *p* < 0.001), increases in sleep quality (B = 0.288, beta = 0.291, *p* < 0.001), increases in total physical activity (B = 0.168, beta = 0.165, *p* = 0.004), as well as decreases in stress experience (B = −0.157, beta = −0.149, *p* = 0.014) The total variance explained by the model as a whole was *R*^2^ = 0.627, *F*(5, 129) = 44.431, *p* < 0.001. The inclusion of the product terms did not explain significant additional variance in the L1 score, corrected *R*^2^ = 0.649, *F*(9, 129) = 27.505, *p* < 0.001, revealing no significant interactions between PH and control group.

Results further showed that the WHO-5 score was significantly lower for the PH group compared to healthy controls (B = −0.511, beta = −0.246, *p* = 0.001). Like for the PH group only, the WHO-5 score was significantly moderated by the individual resilience (B = 0.522, beta = 0.539, *p* < 0.001), subjective stress level (B = −0.179, beta = −0.172, *p* = 0.001), and sleep quality (B = 0.269, beta = 0.278, *p* < 0.001) in the total sample analysis. Total physical activity had also a small significant impact on wellbeing (B = 0.096, beta = 0.097, *p* = 0.044) The total variance explained by the model as a whole was *R*^2^ = 0.740, *F*(5, 127) = 73.393, *p* < 0.001. The inclusion of product terms reflecting interaction by group did not explain significant additional variance in the WHO-5 score, *R*^2^ = 0.738, *F*(9, 127) = 40.809, *p* < 0.001, revealing no significant interactions between PH and control group.

Regarding mental health, results showed that the PHQ-4 score was significantly higher for PH patients compared to healthy controls (B = 0.638, beta = 0.295, *p* < 0.001). Moreover, increases in PHQ-4 scores correlated significantly with decreases in resilience (B = −0.505, beta = −0.504, *p* < 0.001), decreases in sleep quality (B = −0.196, beta = −0.196, *p* = 0.004) and increases in stress (B = 0.235, beta = 0.219, *p* < 0.001). The total variance explained by the model as a whole was *R*^2^ = 0.640, *F*(5, 128) = 46.508, *p* < 0.001. The inclusion of the product terms did explain significant additional variance in the PHQ-4 score, *R*^2^ = 0.685, *F*(9, 128) = 31.85, *p* < 0.001, revealing that significant interactions between group and resilience (B = −0.251, beta = −0.201, *p* = 0.034) as well as group and stress experience (B = 0.299, beta = 0.218, *p* = 0.014) reflecting a lower resilience as well as a higher stress experience during lockdown in patients with PH leading to a stronger rate of change of mental disorders in PH patients (Figure 2C,D).

## 4. Discussion

The present data showed that during the COVID-19 pandemic PH patients had significantly lower physical activity, mental health, and sleep quality compared to age-matched healthy subjects. Being physically active positively correlated with mental health and sleep quality in the PH group. The inclusion of product terms reflecting interaction by group did not explain significant additional variance in the WHO-5 score, revealing no significant interactions between PH and control group. Using multiple regression, data revealed for the PH group that mental health issues could be predicted by total physical activity, sleep, stress level, and resilience indicating that especially resilience, sleep quality and stress experience are relevant predictors of mental health outcomes and wellbeing. The patients’ total physical activity levels, however, only seem to impact life satisfaction. When comparing both groups, we found that in PH patients as well as healthy controls lower resilience, higher stress experience, lower sleep quality as well as reduced physical activity leading to a diminishment of life satisfaction and mental wellbeing. Furthermore, the present data demonstrate that lower resilience and higher stress experience were even leading to a stronger increase of mental illbeing in PH patients compared to healthy controls.

Previous studies documented that PH patients are significantly less active compared to healthy subjects in non-pandemic living conditions [22,23]. During the lockdown period of the pandemic, PH patients seemed to further reduce their activity levels to about 50% compared to accelerometry data from Gonzales-Saiz et al. [22]. Here, one has to keep in mind that accelerometer data are difficult to compare with questionnaire data. However, due to social desirability, most people are even more positive about themselves in surveys than they are [24]. Interestingly, these results are in contrast to recent findings in healthy subjects. Here, it has been demonstrated for a large group of participants that lockdown restrictions did not lead to a decrease in sports activity levels in previously low active subjects [16]. From a therapeutic point of view, this seems to be problematic for PH patients, because a daily activity is an important prognostic factor for the symptomatology and progression of the disease [25].

Concerning mental health issues, PH patients show a significantly decreased mental wellbeing compared to healthy controls. The present data further implicate a prevalence of depression ranging from 36.4% and 53.6% during the pandemic. In contrast, during non-pandemic times, depression prevalence of 7.5 to 55% was reported, with an average prevalence of depression of 36% [5,26,27]. Hence, the prevalence of depressive symptoms of PH patients during the pandemic was found in the upper half of the occurrence under normal circumstances. These findings suggest that social distancing and self-isolation only slightly favor the development of depressive symptoms. However, the more significant factor seems to be the functional limitations of PH patients [26]. Mental impairments that promote depression, are represented by anxious symptoms which were found in about one-third of the PH patients. Compared to the given control group, this prevalence is significantly higher. However, previous studies found anxiety disorders in PH patients in a range of 13 to 45.5% in non-pandemic situations [27,28,29]. Accordingly, the level of anxiety seems to be not significantly higher during the COVID-19 pandemic.

A potential driver of mental disorders seems be the worse sleep quality of PH patients. The self-reported general sleep quality indicates an about 25% lower level compared to subjects of the control group. Similar results were found for the current sleep quality during the COVID-19 pandemic, where the PH group scored 27% lower levels compared to the control group. PH patients seem to suffer also during non-pandemic conditions from a reduced sleep quality compared to healthy subjects [26]. Quality of sleep and the mental health scores WHO-5 and L-1 positively correlated with total physical activity, total activity and climbing stairs. These associations once again underline the close connection between different lifestyle factors. However, it has to be stated that the participation in sports activities is generally low in PH patients, even if there is sufficient evidence available that regular exercise training has an overall positive effect on the physiological and psychological components of PH [25]. The positive relationship between sports activity and mental wellbeing and life satisfaction holds for the PH and the control group as revealed by the missing interaction in the multiple regression analysis which is not surprising given the tremendous evidence for physical activity and sports to promote wellbeing and buffer stress [30]. However, correlations for the control group failed to reach significance. Here, we speculate that this effect is driven by the rather small size of this sub-group.

The present data revealed that especially subjective stress experience as well as individual resilience seem to be strong predictors of mental illbeing of PH patients as well as healthy controls. We assume that subjects of the control group also experience more stress during the pandemic and that their resilience suffers. Therefore, they are approaching the lower level of PH patients. As one major antecedent of mental health issues, like depression, is life stress, both daily hassles [31] and major negative life events [32], it is not surprising that the subjective stress level is strongly associated with mental wellbeing in both groups. Our data revealed that lower resilience and higher stress experience even lead to a stronger increase of mental illbeing in PH patients than in healthy controls reflecting that the present pandemic hit PH patients even harder as their adaptive capacity and resources to react on the situation are lower and mental health might be impaired even more.

The observed impact of resilience on mental health seems plausible and has already been confirmed in healthy subjects [33]. Theory suggests that resilient individuals bounce back from negative experiences quicker and more effectively [34]. As it is demonstrated by a broad body of literature, physical activity is positively associated with a person’s resilience [35]. Recent research showed that especially individuals with high trait anxiety, which may be a risk factor for developing clinically significant mental health problems, may preferentially show psychological, as well as physiological, benefit from physical activity [36]. In the present study, however, this could only partly be confirmed as we found only a small relationship between the activity level and life satisfaction and mental wellbeing.

A limitation of our study is that we used the hemodynamic PH definition of the current European Society of Cardiology (ESC)/European Respiratory Society (ERS) guidelines. The impact of the newly proposed hemodynamic PH criteria on mental health, physical activity, and resilience merit further investigation [2].

## 5. Clinical Implications

Given current research [25] and the present data, we suggest that patients should be encouraged to remain physically active even in pandemic times, although they should certainly do so in consideration of infection control. Vulnerable populations should also receive therapeutic support to improve their sleep quality and stress management as well as psychological resilience factors. All these interventions should also have a positive effect on mental wellbeing accompanied by less anxiety and depressive symptoms [37,38]. Of note, scheduling of appointments of PH patients in specialized outpatient PH expert centers was significantly reduced during the lockdown period [39].

## 6. Conclusions

Current data showed that PH patients showed significantly lower physical activity, mental health, and sleep quality compared to the healthy subjects during the lockdown period of the COVID-19 pandemic. While levels of depression seem to be only slightly affected during the COVID-19 pandemic, significantly lower resilience and higher stress experience lead to an albeit stronger diminishment of wellbeing in PH patients. Hence, it seems desirable to pay special attention to PH patients. Especially in this situation, patients should receive increased therapeutic support to improve lifestyle factors such as sleep quality, stress management and physical activity levels. This could be helpful, for example, in counseling on how to carry out physical activity while observing infection control.

## Figures and Tables

**Table 1:** Sociodemographic characteristics of patients with pulmonary hypertension (PH group) and subjects of the control group.

	Subcategory	PH Group		Control Group	
Age		54.4	± 14.0	52.3	± 8.9
Gender	Female	77.7	(69.6; 84.8)	67.3	(53.8; 80.7)
	Male	22.3	(15.2; 30.4)	32.7	(19.3; 46.2)
Relationship status	Single	19.8	(12.6; 27.9)	11.5	(3.8; 19.2)
	Partnership	68.5	(60.4; 76.6)	86.5	(76.9; 94)
	Other	11.7	(6.3; 18.0)	1.9	(0.0; 5.8)
Living situation 1	Rural	54.1	(45.0; 63.1)	44.2	(30.8; 57.7)
	Suburban	23.4	(15.3; 31.5)	21.2	(11.5; 32.7)
	Urban	22.5	(15.3; 30.6)	34.6	(23.1; 48.1)
Living situation 2	None	6.3	(2.7; 11.7)	7.7	(1.9; 15.4)
	Balcony	23.4	(15.3; 31.5)	25.0	(13.5; 36.5)
	Garden	43.2	(33.3; 53.2)	42.3	(28.8; 57.7)
	Balcony and garden	27.0	(18.9; 35.1)	25.0	(13.5; 36.5)
Residents	Residents per household	2.1	± 1.1	3.0	± 1.3
	Children per household	0.3	± 0.6	0.7	± 1.0
Level of education	Certificate of secondary education	10.1	(4.6; 15.6)	3.8	(0.0; 9.6)
	General certificate of secondary education	18.3	(11.9; 25.7)	5.8	(0.0; 13.5)
	Completed apprenticeship	29.4	(21.2; 38.5)	19.2	(9.6; 30.8)
	Advanced vocational certificate of education	19.3	(11.9; 27.5)	9.6	(1.9; 17.3)
	A level	5.5	(1.8; 10.1)	17.3	(7.7; 28.8)
	Bachelor’s degree	8.3	(3.7; 13.8)	3.8	(0.0; 9.6)
	Master’s degree	9.2	(4.6; 14.7)	30.8	(19.2; 42.3)
	PhD	0		9.6	(1.9; 19.2)
Occupational status	Student/articled	0.9	(0.0; 2.8)	0	
	Full-time equivalent	19.8	(12.3; 27.4)	46.2	(32.7; 59.6)
	Half-time equivalent	7.5	(2.8; 13.2)	7.7	(1.9; 15.4)
	Public official	0.9	(0.0; 2.8)	13.5	(5.8; 23.1)
	Self-employed	2.8	(0.0; 6.6)	9.6	(1.9; 17.3)
	Unemployed	1.9	(0.0; 4.7)	0	
	Retired	45.3	(35.8; 55.6)	15.4	(5.8; 25.0)
	Other	20.8	(13.2; 28.3)	7.7	(1.9; 15.4)
Comorbidities		2.61	± 2.0	0	± 0

Data are presented as an arithmetic mean ± SD or % (n/N) and (95%-CI). The PH group is lacking data >2% in four variables: children per household (6), level of education (3), occupational status (6), comorbidities (3). There were no missing cases in the control group regarding the listed variables.

**Table 2:** Characteristics and group differences of subjects of the PH and control group.

Variable	Subcategory	PH Group		Control Group		*p*-Value
Physical activity	Activity of daily living (ADL) (min/week)	551.4	±816.9	707.0	±686.8	<0.001
	Sports activity (min/week)	129.4	±219.8	407.5	±314.7	<0.001
	Total physical activity (min/week)	684.3	±954.3	1103.8	±851.4	<0.001
	Climbing stairs (floors/week)	14.3	±23.0	38.0	±56.6	<0.001
Mental health	WHO-5	11.9	±5.4	16.0	±4.1	<0.001
	PHQ-4	3.9	±3.0	1.6	±1.7	<0.001
	L-1	5.6	±2.7	7.5	±1.6	<0.001
Resilience (z-standard.)		−0.2	±0.85	0.0	±0.88	n.s.
Stress (z-standard.)		0.0	±1.01	0.0	±1.0	n.s.
Support of surrounding people	Respected	2.8	±1.1	3.3	±0.6	0.002
	Supported	2.9	±1.0	3.2	±0.7	n.s.
	Liked	3.1	±1.0	3.3	±0.7	n.s.
	Sum	8.7	±2.7	9.8	±1.7	n.s.
Sleep	General sleep quality	5.7	±2.4	7.5	±1.8	<0.001
	Acute sleep quality	5.3	±2.5	7.3	±1.9	<0.001
Lifestyle estimation	Satisfaction with nutrition	3.8	±1.7	4.3	±1.3	n.s.
	Worries about health	4.5	±1.6	2.5	±1.7	<0.001
	Satisfaction with sports behavior	2.3	±1.8	3.9	±1.6	<0.001
	Worries about finances	2.4	±2.3	1.8	±1.6	n.s.

Data are presented as the arithmetic mean ± SD. *p*-values were adjusted to *p* < 0.0026 through the level for multiple comparisons. WHO-5: The World Health Organization-Five Well-Being Index, PHQ-4: Patient Health Questionnaire-4; L-1: Likert-scale rating-1.

**Table 3:** Spearman’s rank correlation coefficients for associations between activity scores, sociodemographic data, mental health, and sleep quality of PH patients. Significant associations are depicted in bold.

	Activity of Daily Living		Sports Activity		Total Physical Activity		Climbing Stairs	
Age	−0.19	(0.080)	−0.23	(0.020)	−0.22	(0.041)	0.22	(0.041)
Level of education	0.06	(0.570)	0.27	(0.007)	0.14	(0.202)	0.06	(0.575)
Residents per household	0.20	(0.052)	0.09	(0.353)	0.20	(0.067)	0.21	(0.049)
Children per household	0.18	(0.101)	−0.00	(0.945)	0.19	(0.093)	0.19	(0.080)
Comorbidities	−0.20	(0.062)	0.03	(0.787)	−0.17	(0.116)	0.13	(0.234)
WHO-5	0.33	(0.002)	0.09	(0.396)	0.26	(0.016)	0.33	(0.002)
PHQ-4	−0.16	(0.130)	0.13	(0.207)	−0.11	(0.300)	0.23	(0.031)
L-1	0.33	(0.001)	0.04	(0.716)	0.30	(0.005)	0.31	(0.003)
General sleep quality	0.33	(0.001)	0.11	(0.263)	0.31	(0.003)	0.39	(<0.001)
Acute sleep quality	0.32	(0.002)	0.09	(0.385)	0.25	(0.018)	0.32	(0.002)
Resilience (z-standard.)	0.24	(0.027)	0.058	(0.572)	0.20	(0.076)	0.31	(0.004)
Stress (z-standard.)	0.06	(0.578)	0.074	(0.461)	0.85	(0.438)	0.05	(0.649)

WHO-5: The World Health Organization-Five Well-Being Index, PHQ-4: Patient Health Questionnaire-4; L-1: Likert-scale rating-1.

## Data Availability

All data are available upon request.
